# Supplementary material for: Allosteric regulation and crystallographic fragment screening of SARS-CoV-2 NSP15 endoribonuclease
Source: Nucleic Acids Res. 2023 Apr 28;51(10):5255–70. doi: 10.1093/nar/gkad314 (PMC10250223; doi:10.1093/nar/gkad314)
Supplement: gkad314_Supplemental_Files [file gkad314_supplemental_files.zip › Supplementary datasets.zip!/224/Refine_9/01-BUSTER/Cycle-4/shell.01/R_Rfree.html]

# Rxpct factors in resolution bins at cycle 0


|  |  |  |  |  |  |  |  |  |
| --- | --- | --- | --- | --- | --- | --- | --- | --- |
|  | **<d> (Å)**  Rxpct\_Work | Rxpct\_Free | Rxpct\_All | |  |  |  |  |
|  | 15.0574 | 0.3671 | 0.5158 | 0.3722 |  |
|  | 8.6934 | 0.2360 | 0.2647 | 0.2377 |  |
|  | 6.7339 | 0.2258 | 0.2461 | 0.2270 |  |
|  | 5.6912 | 0.2129 | 0.2712 | 0.2148 |  |
|  | 5.0191 | 0.1752 | 0.2247 | 0.1782 |  |
|  | 4.5400 | 0.1629 | 0.1861 | 0.1641 |  |
|  | 4.1762 | 0.1705 | 0.1942 | 0.1718 |  |
|  | 3.8878 | 0.1782 | 0.2295 | 0.1805 |  |
|  | 3.6520 | 0.1822 | 0.2317 | 0.1846 |  |
|  | 3.4544 | 0.1814 | 0.2275 | 0.1841 |  |
|  | 3.2858 | 0.1898 | 0.2343 | 0.1924 |  |
|  | 3.1397 | 0.1910 | 0.2122 | 0.1920 |  |
|  | 3.0115 | 0.1903 | 0.2146 | 0.1914 |  |
|  | 2.8978 | 0.1994 | 0.2029 | 0.1996 |  |
|  | 2.7961 | 0.1994 | 0.2380 | 0.2012 |  |
|  | 2.7044 | 0.2052 | 0.2304 | 0.2065 |  |
|  | 2.6212 | 0.2050 | 0.2338 | 0.2067 |  |
|  | 2.5452 | 0.2197 | 0.2509 | 0.2214 |  |
|  | 2.4754 | 0.2245 | 0.3011 | 0.2290 |  |
|  | 2.4111 | 0.2473 | 0.2729 | 0.2484 |  |
|  | **Overall** | 0.1996 | 0.2354 | 0.2014 |  |

# Rxpct factors in resolution bins at cycle 1


|  |  |  |  |  |  |  |  |  |
| --- | --- | --- | --- | --- | --- | --- | --- | --- |
|  | **<d> (Å)**  Rxpct\_Work | Rxpct\_Free | Rxpct\_All | |  |  |  |  |
|  | 15.0574 | 0.3673 | 0.5161 | 0.3724 |  |
|  | 8.6934 | 0.2361 | 0.2648 | 0.2378 |  |
|  | 6.7339 | 0.2255 | 0.2457 | 0.2266 |  |
|  | 5.6912 | 0.2122 | 0.2707 | 0.2141 |  |
|  | 5.0191 | 0.1746 | 0.2246 | 0.1776 |  |
|  | 4.5400 | 0.1622 | 0.1857 | 0.1634 |  |
|  | 4.1762 | 0.1697 | 0.1940 | 0.1711 |  |
|  | 3.8878 | 0.1773 | 0.2291 | 0.1796 |  |
|  | 3.6520 | 0.1814 | 0.2316 | 0.1838 |  |
|  | 3.4544 | 0.1806 | 0.2268 | 0.1833 |  |
|  | 3.2858 | 0.1891 | 0.2340 | 0.1917 |  |
|  | 3.1397 | 0.1904 | 0.2124 | 0.1914 |  |
|  | 3.0115 | 0.1900 | 0.2152 | 0.1911 |  |
|  | 2.8978 | 0.1993 | 0.2032 | 0.1995 |  |
|  | 2.7961 | 0.1994 | 0.2381 | 0.2012 |  |
|  | 2.7044 | 0.2049 | 0.2305 | 0.2062 |  |
|  | 2.6212 | 0.2048 | 0.2341 | 0.2066 |  |
|  | 2.5452 | 0.2193 | 0.2508 | 0.2211 |  |
|  | 2.4754 | 0.2240 | 0.3009 | 0.2285 |  |
|  | 2.4111 | 0.2468 | 0.2731 | 0.2479 |  |
|  | **Overall** | 0.1990 | 0.2353 | 0.2009 |  |

# Rxpct factors in resolution bins at cycle 2


|  |  |  |  |  |  |  |  |  |
| --- | --- | --- | --- | --- | --- | --- | --- | --- |
|  | **<d> (Å)**  Rxpct\_Work | Rxpct\_Free | Rxpct\_All | |  |  |  |  |
|  | 15.0574 | 0.3675 | 0.5161 | 0.3726 |  |
|  | 8.6934 | 0.2361 | 0.2648 | 0.2377 |  |
|  | 6.7339 | 0.2250 | 0.2452 | 0.2261 |  |
|  | 5.6912 | 0.2113 | 0.2700 | 0.2133 |  |
|  | 5.0191 | 0.1738 | 0.2246 | 0.1769 |  |
|  | 4.5400 | 0.1614 | 0.1852 | 0.1626 |  |
|  | 4.1762 | 0.1689 | 0.1937 | 0.1703 |  |
|  | 3.8878 | 0.1763 | 0.2287 | 0.1787 |  |
|  | 3.6520 | 0.1807 | 0.2314 | 0.1831 |  |
|  | 3.4544 | 0.1799 | 0.2263 | 0.1826 |  |
|  | 3.2858 | 0.1885 | 0.2338 | 0.1911 |  |
|  | 3.1397 | 0.1899 | 0.2128 | 0.1909 |  |
|  | 3.0115 | 0.1897 | 0.2155 | 0.1909 |  |
|  | 2.8978 | 0.1991 | 0.2034 | 0.1993 |  |
|  | 2.7961 | 0.1994 | 0.2381 | 0.2012 |  |
|  | 2.7044 | 0.2047 | 0.2308 | 0.2061 |  |
|  | 2.6212 | 0.2048 | 0.2345 | 0.2066 |  |
|  | 2.5452 | 0.2192 | 0.2510 | 0.2210 |  |
|  | 2.4754 | 0.2239 | 0.3008 | 0.2284 |  |
|  | 2.4111 | 0.2464 | 0.2735 | 0.2476 |  |
|  | **Overall** | 0.1985 | 0.2351 | 0.2004 |  |

# Rxpct factors in resolution bins at cycle 3


|  |  |  |  |  |  |  |  |  |
| --- | --- | --- | --- | --- | --- | --- | --- | --- |
|  | **<d> (Å)**  Rxpct\_Work | Rxpct\_Free | Rxpct\_All | |  |  |  |  |
|  | 15.0574 | 0.3799 | 0.5217 | 0.3848 |  |
|  | 8.6934 | 0.2298 | 0.2778 | 0.2326 |  |
|  | 6.7339 | 0.2071 | 0.2410 | 0.2091 |  |
|  | 5.6912 | 0.1841 | 0.2266 | 0.1855 |  |
|  | 5.0191 | 0.1492 | 0.2107 | 0.1530 |  |
|  | 4.5400 | 0.1350 | 0.1752 | 0.1370 |  |
|  | 4.1762 | 0.1403 | 0.1739 | 0.1422 |  |
|  | 3.8878 | 0.1491 | 0.2131 | 0.1520 |  |
|  | 3.6520 | 0.1584 | 0.2212 | 0.1614 |  |
|  | 3.4544 | 0.1608 | 0.2028 | 0.1633 |  |
|  | 3.2858 | 0.1733 | 0.2193 | 0.1760 |  |
|  | 3.1397 | 0.1785 | 0.2167 | 0.1802 |  |
|  | 3.0115 | 0.1847 | 0.2240 | 0.1865 |  |
|  | 2.8978 | 0.1981 | 0.2168 | 0.1991 |  |
|  | 2.7961 | 0.2024 | 0.2485 | 0.2045 |  |
|  | 2.7044 | 0.2083 | 0.2432 | 0.2101 |  |
|  | 2.6212 | 0.2093 | 0.2471 | 0.2116 |  |
|  | 2.5452 | 0.2262 | 0.2667 | 0.2285 |  |
|  | 2.4754 | 0.2352 | 0.2948 | 0.2387 |  |
|  | 2.4111 | 0.2582 | 0.3031 | 0.2601 |  |
|  | **Overall** | 0.1840 | 0.2287 | 0.1863 |  |
